# Scraps

**Published:** 1890-09

**Authors:** 


### SCRAPS

PICKED UP BY THE ASSISTANT-EDITOR.

---

**"The Turn of Life."**—A subscriber to the *Journal* tells me that recently in a form of proposal for life-insurance the following was given as one portion of the applicant's family history:

| | |
|---|---|
| Original number of sisters . . . | *1.* |
| Age at death . . . . . . . . . | *55 about.* |
| Cause of death . . . . . . . . . | *Eternal life. Dr. S****** attended her.* |

**The Xth International Medical Congress.**—A local doctor just back from the Congress tells me that a Berlin paper contained the following "Programme of Papers in the Special Department." He does not think, however, that any of the papers were read.

| | |
|---|---|
| Prof. Dr. VON MOLTKE: | "On the bleeding which may be applied to whole nations." |
| Prof. MIGUEL: | "On the heaping up of gold molecules in the arteries of States." |
| Dr. BRÖMEL: | "On the bad results of the iron cure in international diseases." |
| Prof. Dr. BISMARCK: | "On urgency of speech as a symptom which follows vanishing omnipotence." |
| Dr. SALISBURY: | "On the colonial fever." |
| Dr. OFFICIOSUS: | "On removal of sleeplessness by the use of the *Imperial Gazette.*" |
| Prof. LESSEPS: | "On the antiseptic resection of isthmuses and the cupping of shareholders." |
| Dr. MILAN: | "On the central disturbances which occur when one takes off the crown in a hurry." |

**Longevity.**—In connection with the pseudo-centenarianism of the Elgin "Methuselah," whose death was recently recorded (*Pall Mall Gazette*, August 26th), the following story will be read with interest:—It is well known that people seldom die in Ochiltree. When any one there gets tired of life, he generally finds it necessary to remove to another parish. Many of the inhabitants however do not seem ever to tire of life, and so remain on their native soil till their ages are past all reckoning. Colonel Andrew M'Dowall, when he returned from India, came one day upon an old man sitting weeping on a big stone by the roadside. When he came up, the old man rose and took off his bonnet and wiped his eyes, and said, "Ye're welcome hame again, laird." "Thank you," said the Colonel, adding, after a pause, "I should scarcely know your face; aren't you Nathan M'Culloch?" "Ye're richt, 'deed!" said Nathan. "It's just me, laird." "You must be a good age now, Nathan," said the Colonel. "I'm no verra aul' yet, laird," was the reply; "I'm just turn't a hunner." "A hundred!" said the Colonel, musing. "Well, you must be all that. But the idea of a man of a hundred sitting blubbering in that way! What could you get to cry about?" "It was my faither licket me, sir," said Nathan, weeping again, "an' he put me oot, so he did!" "Your father!" said the Colonel, astonished. "Is your father alive yet?" "Leevin'?

Ay," replied Nathan, "I ken that the day tae my sorrow." "Where is he?" said the Colonel. "What an age he must be! I should like to see him." "Oh, he 's up in the barn there," said Nathan, "an' no in a horrid gude humour the noo aither!" They went up to the farm together, and found the father busy threshing the barley with the big flail and going on at a fearful rate. Seeing Nathan and the laird coming in, he stopped and saluted the Colonel, who, after inquiring how he was, asked him what he had struck Nathan for. "The young rascal," said the father, "there 's nae dooin' wi' him; he 's never oot o' mischief! I had tae lick him this mornin' for cloddin' stanes at his grandfather!"

**Anatomy and the Etymologists.**—E. R. P., writing to the *Journal of Cutaneous and Genito-Urinary Diseases*, said: "A member of the Bar, distinguished alike for his eminence as a jurist and his profound general erudition, gave me recently the etymology of a familiar word that few can correctly spell and still fewer trace to its origin. He said the Roman diminutive *ette* contracted to *et* abounds in such words as streamlet, a little stream; rivulet, a little river, etc.; while the Saxon diminutive *ock* serves a similar purpose in such words as a hillock, a little hill, and bullock, a little bull, and plural, bullocks, little bulls. So it appears at last that the familiar name by which the schoolboy calls his testicles does not belong to the realm of slang, but descends to us straight from the shelves of mediæval classics." Benjamin Lee, commenting upon this, observed that to make the vulgar name for the testicles identical with bullocks "involves an error in orthography and is also entirely inadequate. The word as in common use is spelled correctly with 'o,' not 'u.' The true descent of the word is from 'boll,' which means a seed-vessel; whence, naturally enough, 'bollocks,' little seed-vessels. It is Saxon, pure and undefiled, and not a vulgar play upon words; and, moreover, it is strictly and scientifically correct." Neither of these writers is quite accurate. Readers of the Old Testament are familiar with the passage, "The barley was in the ear, and the flax was bolled" (*Exodus* ix. 31), where the word means "podded for seed." But the root goes further back than this meaning, which was a derived one. As Aldis Wright says *(Bible Word-Book)*, the word is etymologically connected with ball, etc., and the root expresses the idea of roundness, swelling. In connection with this, these extracts from the *Catholicon Anglicum*, "an English-Latin Wordbook, dated 1483," will be of interest: "a Balloke stone; *testicul*us, *testiculat*us *part*icipium; a Ballokecod; *piga*, *im*mb*rana*;" to which Mr. Sidney J. H. Herrtage, [the editor of the Camden Society edition (1882) of the *Catholicon*, appends this note: "'*Hic testiculus*, a balok-ston; *hic piga*, a balok-kod,' Nominale MS. 15th cent. '*Couille*, a cod, bollock, or testicle,' Cotgrave. It appears from Palsgrave's Acolastus, 1540, that *ballocke-stones* was a term of endearment." But the instance from Cotgrave is wrongly cited. At least, in the 1632 edition it is given thus: "Couille: f. *A mans yard; also (but lesse properly) the cod, ballocke, or testicle* . . ." and I have not found "bollock" anywhere. As the English equivalent of "Couillon de chien," Cotgrave gives "Balock-grasse" along with "Dogs-stones," and other popular names of several species of orchis. The *New English Dictionary* has under "Ballock" several quotations of varying forms of the word, of which one of the most interesting is from *Leviticus* xxii. 24, where Wyclif read "kitt and taken awey the ballokes is," which the A.V. renders merely as "cut." (For "kitt" as "cut" see quotations from Wyclif in Richardson's *Dictionary*, under "cut.") From all which it is clear that "the familiar name by which the schoolboy calls his testicles" should be spelled "ballocks."

